# Infrequent Placental and Fetal Involvement in SARS-CoV-2 Infection: Pathology Data from a Large Medical Center

**DOI:** 10.3390/jdb9040045

**Published:** 2021-10-16

**Authors:** Jeffrey Thomas, Yu Sun, Larisa Debelenko

**Affiliations:** Department of Pathology and Cell Biology, Columbia University—Irving Medical Center, New York, NY 10032, USA; jt3236@cumc.columbia.edu (J.T.); ys3271@cumc.columbia.edu (Y.S.)

**Keywords:** SARS-CoV-2, IHC, ISH, pregnancy, placenta, intervillositis, autopsy, perinatal, fetal demise

## Abstract

In order to determine the frequency of SARS-CoV-2 placental and fetal involvements, we analyzed placentas of 197 women positive for infection at delivery and fetal tissues in cases of pregnancy loss in women positive by SARS-CoV-2 PCR (*N* = 2) and COVID-19 serology (*N* = 4), using in situ hybridization (ISH), immunohistochemistry (IHC) and, in selected cases, RT-PCR of tissue homogenates. The virus was identified in situ, accompanied by intervillositis, in 2 of 197 placentas (1.02%). In three more cases, SARS-CoV-2 was detected by tissue PCR without in situ localization and placental inflammation. There were no maternal mortality or association of placental infection with the clinical severity of COVID-19. All tested neonates born to SARS-CoV-2-positive women (*N* = 172) were negative for the virus. There were three pregnancy losses among 197 infected women and in two cases available fetal tissues were negative for SARS-CoV-2. In one of four fetal autopsies performed in women with positive COVID-19 serology, the mother-to-child transmission (MTCT) could be inferred based on positive SARS-CoV-2 nucleocapsid IHC in fetal pulmonary endothelium. Placental involvement by SARS-CoV-2 is rare, but may be underestimated due to its transient nature. MTCT is even rarer, supporting the protective role of placenta in SARS-CoV-2 infection.

## 1. Introduction

According to the recent international retrospective cohort study (PregOuTCOV), 3.6% of pregnant women have been infected by SARS-CoV-2 during pregnancy and the viral exposure was associated with composite adverse obstetric and neonatal outcomes, which prompted the authors to advocate for vaccination before or early in pregnancy [1]. Furthermore, a multinational cohort study established increased risks of maternal and neonatal morbidity and mortality indices in women with COVID-19 diagnosis compared to women without COVID-19 diagnosis [2].

Placental involvement by SARS-CoV-2 has been documented by numerous case reports [3,4,5,6,7,8,9,10] with the earlier cases recently summarized [11,12] to outline characteristic histomorphologic features of SARS-CoV-2 placental infection. The NIH placenta workshop established a classification scheme and criteria for the placental involvement depending on technologies used for the viral detection [13]; however, the frequency of placental infection in COVID-19 remains unknown.

The question of transplacental or mother-to-child transmission (MTCT) of SARS-CoV-2 is complicated with several proposed classification schemes and different sets of data analyzed by different methodologies; however, according to recent reviews, MTCT of SARS-CoV-2 is possible, although, similar to the placental involvement, its incidence and effects on the newborn have not been elucidated [14,15].

The role of COVID-19 infection in pregnancy loss is even less clear; however, a retrospective cohort study using a database of pregnant people giving birth in Ontario, Canada, between July 2002 and December 2020 did not find any cause variation (unusual pattern) in preterm birth or stillbirth rates during the first 12 months of COVID-19 pandemic compared with the previous 17.5 years [16].

We analyzed placentas of women infected with SARS-CoV-2 using ISH and ICH in order to determine the frequency of placental/transplacental infection and its association with the disease severity. Fetal tissues from perinatal autopsies in infected women were also analyzed in order to identify cases of MTCT and its possible role in perinatal loss. This analysis summarizes all our cases during the 1st year of the COVID-19 epidemic starting from 15 March 2020, including subsets of cases published by us previously [7,17,18,19].

## 2. Materials and Methods

The study was approved by the Institutional Review Board (Protocol IRB-AAAT0272). We performed a retrospective review of placental pathology and neonatal autopsy reports and pathology samples from the files of our Pathology Department. Pertinent clinical information was extracted from the medical records. The waver of the patients’ consent was granted based on the criteria which governed the study design, including an adequate plan to protect subjects’ personal information (coding, secure handling) and destroy the patients’ identifiers after the study completion, preventing reuse or disclosure of the personal information. We analyzed clinical information of 1950 women who gave birth during one year since the start of COVID-19 epidemic in March of 2020 and whose placental specimens were available. We included placentas of 197 women who tested positive for SARS-CoV-2 by the nasopharyngeal swab RT-PCR test on admission for labor and delivery during the period from 15 March 2020 to 14 March 2021. Additionally, we analyzed placental pathology of 84 women who had negative PCR, but positive COVID-19 serology tests during the first quarter of 2021. We also studied fetal tissues in 6 cases of perinatal autopsies performed during the same period of 2020/21 if mothers had positive RT-PCR (N = 2) or COVID-19 serology (N = 4) results.

SARS-CoV-2 IHC and ISH were performed on paracentral full length sections of placental disc using probes, antibodies, instruments, and reagents as previously described [7]. Placental tissue was refrigerated after delivery and fixed in 10% buffered formaldehyde no later than 24 h after delivery, as recommended by the NIH workshop [13]. One hundred fifty three of 197 (77.7%) placentas of SARS-CoV-2 positive women were studied with combinations of ISH with a sense probe complementary to viral sequences encoding for the spike protein and IHC with anti-nucleocapsid and anti-spike antiviral antibodies. Tissue sections of fetal/neonatal lungs in cases of positive maternal testing (*N* = 6) were also studied with SARS-CoV-2 ISH and IHC and in 3 cases the viral RT-PCRs of homogenates of the lung tissue were performed as previously described [7].

Placental involvement was defined as definitive in the presence of (1) intervillositis (placentitis) with villous trophoblast necrosis, (2) syncytiotrophoblast positivity for SARS-CoV-2 by ISH and ICH [11,12,13]. Placental involvement was defined as possible in the presence of positive SARS-CoV-2 RT-PCR of tissue homogenates but negative SARS-CoV-2 ISH and IHC and lack of the inflammation in the placental tissue.

## 3. Results

### 3.1. Maternal and Placental Infection

**Figure 1:**
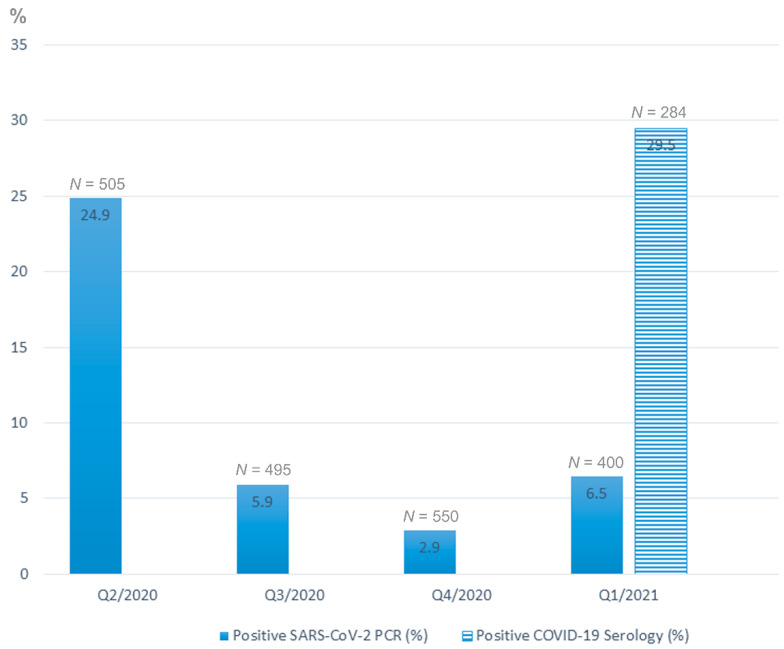
Dynamics of SARS-CoV-2 infection in parturient women in our medical center.

One hundred ninety seven of 1950 (10.1%) women who gave birth between 15 March 2020 and 14 March 2021 tested positive for SARS-CoV-2 by RT-PCR of nasopharyngeal swab within 24 h of delivery, including 126 of 505 (24.9%) in the second, 29 of 495 (5.9%) in the third, 16 of 550 (2.9%) in the fourth quarters of 2020, and 26 of 400 (6.5%) in the first quarter of 2021 (Figure 1). In the first quarter of 2021, the data on COVID-19 serology became available and showed that 84 of 284 (29.5%) women were positive for this test, while their RT-PCRs were negative, both taken within 24 h of delivery (Figure 1).

The infection was detected by nasopharyngeal swab RT-PCR tests from the second quarter of 2020 (Q2/2020) to the first quarter of 2021 (Q1/2021). COVID-19 serology tests results available for Q1/2021 are also presented. *N*—numbers of studied placentas with available SARS-CoV-2 testing results in each quarter. The majority of infected women were either asymptomatic or post symptomatic at the time of delivery; however, 21% (42 of 197) reported symptoms of COVID-19, including six women who had a severe disease, requiring mechanical ventilation in five and extracorporeal oxygenation (ECMO) in one. There was, however, no maternal mortality in the studied group.

The definitive placental involvement by SARS-CoV-2 was seen in 2 of 197 (1.02%) placentas including one previously reported case [7].

**Figure 2:**
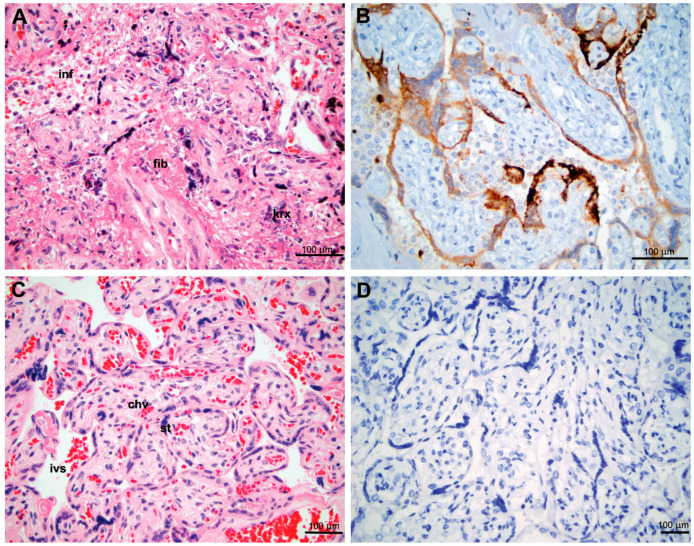
Histopathology and immunohistochemistry of a definitive placental involvement by SARS-CoV-2 and age-matched placental control (gestational age: 39 weeks). (**A**) Intervillositis with mild inflammatory infiltrate in intervillous spaces (inf), massive necrosis of syncytiotrophoblast with karyorrhexis (krx) and perivillous deposition of eosinophilic fibrinoid proteins (fib). Hematoxilin and Eosin (H&E). (**B**) IHC with the antibody against the SARS-CoV-2 nucleocapsid protein positive in syncytiotrophoblast covering chorionic villi and monocytes scattered in intervillous spaces. (**C**) Histology of an unaffected placenta at the same gestational age (39 weeks), showing chorionic villi (chv) covered with syncytiotrophoblast (st) and intact intervillous spaces (ivs) with red blood cells. H&E. (**D**) Negative IHC with antibody against the SARS-CoV-2 nucleocapsid protein in the unaffected placenta.

The second case presented in November 2020 and involved a 46 year old Gravida 2 Para1011 who had an induction of labor at 39 4/7 weeks and forceps-assisted vaginal delivery. She was SARS-CoV-2 RT-PCR-positive with mild symptoms at the day of delivery with negative COVID-10 serology and negative RT-PCR 10 days prior. The antenatal history was notable for travel-induced deep vein thrombosis. The newborn girl had Apgar scores 9 and 9 at 1 and 5 min, respectively, and birth weight of 3125 g (25–50th percentile). The girl was healthy-appearing, vigorous, and passed all newborn screens except for an elevated transcutaneous bilirubin. Her SARS-CoV-2 RT-PCR test was negative, and the mother and the child were discharged on hospital day 2 in good health. The follow up visits were unremarkable. Placental pathology included patchy intervillous inflammation with histiocytes (macrophages) as a predominant cell type; this intervillositis was accompanied by necrosis of villous trophoblast with marked karyorrhexis and perivillous accumulation of fibrinoid proteins (Figure 2A). IHC for SARS-CoV-2 was positive in the syncytiotrophoblast lining the chorionic villi (Figure 2B).

None of the remaining 195 placentas of infected women showed a constellation of findings diagnostic of a definitive SARS-CoV-2 involvement; however, three cases that were reported by us previously [18,19] were consistent with a possible involvement based on positive RT-PCR of homogenized placental tissue not accompanied by the placental inflammation and staining with antiviral IHC and ISH.

**Figure 3:**
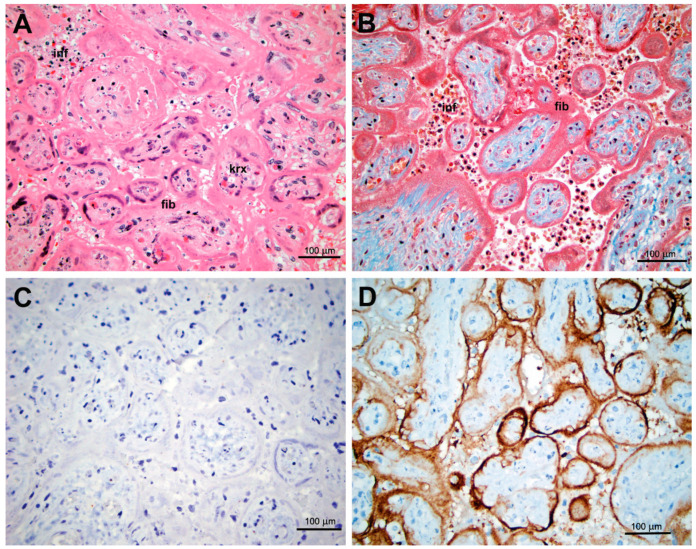
Histopathology and immunohistochemistry of placenta in a parturient woman with severe COVID-19 (gestational age: 24 4/7 weeks). (**A**) Intervillositis with scant inflammatory infiltrate (inf) with karyorrhexis, trophoblast necrosis and dense eosinophilic fibrinoid (fib) material enveloping chorionic villi. Villous stromal karyorrhexis (krx), consistent with hypoxia, is also seen. H&E. (**B**) Intervillositis (inf) with trophoblast necrosis, fibrinoid material (fib), enveloping chorionic villi. Trichrome stain. (**C**) Negative IHC with antibody against the SARS-CoV-2 nucleocapsid protein. (**D**) IHC with antibody against complement complex C4d highlighting the interface complement accumulation and necrotic syncytiotrophoblast.

Intervillositis, an unspecific inflammatory placental lesion associated with SARS-CoV-2 as well as with some other infectious and non-infectious etiologies, was observed in 10 additional cases (5.1%) in the group of positive women without concurrent tissue positivity for SARS-CoV-2 by ICH or ISH. One of these 10 cases involved a previously healthy 36 year old Gravida 2 Para 1001 woman in a critical condition due to severe COVID-19 complicated by cardiomyopathy and acute kidney and respiratory failure, necessitating ECMO placement. Because of the non-reassuring fetal heart rate tracing, the decision was made to deliver at 24 4/7 weeks by Cesarean section. The baby boy was born with Apgar scores 3 and 7 at 1 and 5 min, respectively, and a low birthweight of 990 g. He developed severe conditions associated with systemic hypoxia and prematurity (grade 1 germinal matrix hemorrhage and moderate respiratory distress, requiring intubation); however, his SARS-CoV-2 RT-PCR studies were negative. The mother fully recovered within a course of several weeks; the child was on ventilation support for 2 months, but eventually was weaned off and discharged with persistent pulmonary hypertension. Placental histopathology showed patchy areas of a mild intervillositis with marked necrosis of syncytiotrophoblast, highlighted by the complement component C4d and a dense layer of perivillous fibrin enveloping affected chorionic villi with focally ischemic cores; however, antiviral ISH and IHC were negative (Figure 3).

Placentas of five remaining women with severe COVID-19 at delivery which were reported previously [19] did not show acute changes attributable to infection; however, in one of these cases the viral RNA was amplified by RT-PCR of placental tissue homogenates, consistent with a possible placental involvement [13].

In the group of 84 women presented with negative viral RT-PCR but positive serology tests, intervillositis was diagnosed in four cases (4.8%), all negative for SARS-CoV-2 by IHC.

### 3.2. Fetal and Neonatal Involvement

One hundred seventy-two neonates born to the one hundred and ninety-seven mothers positive for the virus (87.3%) were tested for SARS-CoV-2 by nasopharyngeal swab RT-PCRs within the first 48 h of life and all test results were negative. There were three cases of fetal/neonatal loss in the group of 197 SARS-CoV-2 positive women, including one early second trimester miscarriage, one intrauterine fetal demise (IUFD), and one early neonatal death. In two of these three cases reported by us previously [18], fetal tissues were negative for the virus by RT-PCR, IHC, and ISH and the causes of IUFD were defined as not related to COVID-19 (chronic deciduitis in one case and acute ascending intrauterine infection with group B strep in the second). The third case involved an IUFD at 28 weeks in a 34 year old Gravida 4 Para 3103 women with the history of systemic lupus erythematosus. Fetal ultrasound showed corpus callosum agenesis with ventriculomegaly and grey matter heterotopia. The autopsy was not requested.

During the 1st year of the COVID-19 epidemic, we performed 35 autopsies of stillborn and live born babies whose maternal SARS-CoV-2 PCR status was known and, besides the two cases outlined in the previous paragraph, there were no additional perinatal autopsies from mothers infected with the virus at the time of delivery.

**Figure 4:**
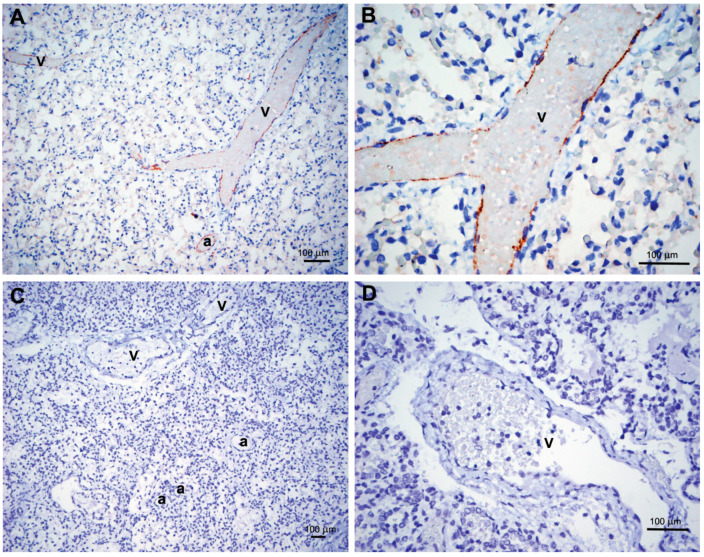
Immunohistochemistry with antibody against SARS-CoV-2 nucleocapsid protein in lungs of 3 stillborn fetuses of mothers with positive COVID-19 serology testing. (**A**,**B**) Positive punctate and granular staining of the endothelium of distended veins (v) and small arteries (a) in a 38-week fetus. (**C**) Negative staining results in a 26-week fetus. (**D**) Negative staining results in a 30-week fetus.

Maternal COVID-19 serology results available in 17 of 35 perinatal autopsies were positive in four cases (23.5%). In three cases the causes of death included a cord accident (true umbilical cord knot), immune hydrops in Rh- mother, and non-immune hydrops with marked pulmonary hypoplasia. In the fourth case of a near term (38 weeks) pregnancy loss to a 27 year old multigravida with a high body/mass index (35.4), the cause of IUFD was not apparent. Placental and fetal tissues in the first three cases showed no signs of inflammation or immunoreactivity with anti-spike or anti-nucleocapsid SARS-CoV-2 antibodies. In the fourth case, the placenta was negative for the virus; however, an endothelial staining with anti-nucleocapsid, but not anti-spike, antibody was present in the fetal lungs (Figure 4A,B). SARS-CoV-2 ISH and RT-PCR from pulmonary tissue homogenates were, however, negative for replicating viruses. Interestingly, the neonatal blood was also positive for COVID-19 serology.

## 4. Discussion

The data on the prevalence of COVID-19 infection among parturient women in our medical center followed the temporal dynamics of the epidemic in New York City with its peak in the second quarter of 2020, a subsequent drop in the second half of 2020, and a mild gradual increase in the first quarter of 2021. Interestingly, that near one third (29.5%) of placentas submitted for pathology evaluation in the first quarter of 2021 came from women with positive COVID-19 serology tests. The COVID-19 serology test employed by our medical center uses the ROCHE immunoassay intended for a qualitative detection of antibodies to SARS-CoV-2 under the Emergency Use Authorization and should be interpreted as an evidence of prior infection, according to the Center for Disease Control (CDC) recommendations. Thus, the number 29.5% reflects the prevalence of prior COVID-19 in the group of pregnant women with high risk and complicated pregnancies whose placentas have been submitted for pathological evaluation, according to the institutional policies, and should not be extrapolated to the general population.

Our data also indicate that although 3% of women infected with SARS-CoV-2 had severe disease at the time of delivery there were no cases of maternal mortality among symptomatic (as well as asymptomatic) patients and no fetal mortality in the group of severely ill. In one case, however, the severe COVID-19 disease prompted the delivery at 24+ weeks, leading to a significant fetal morbidity related to prematurity.

To assess the frequency of definitive placental involvement, we used recently proposed criteria [11,12,13] which include: (1) placental inflammation (intervillositis); (2) necrosis of villous trophoblast with perivillous accumulation of fibrillary proteins; (3) in situ identification of replicating virus in villous trophoblast. Thus, definitive placental involvement was identified only in two patients (about 1% in our cohort).

Although in the group of six severely ill patients there were no definitive placental infection, in one case, reported by us previously [19], positive RT-PCR results from placental homogenates were consistent with a possible involvement. In this case, an elevated expression of interferon-induced antiviral transmembrane transcripts was also detected by molecular techniques while no tissue reaction (inflammation, necrosis) was observed microscopically. This demonstrates that before the morphological changes become apparent the infection may develop on the molecular level and if delivery occurs in early stages of the disease placental lesions may not be detectable on the microscopic level.

Similarly, in the late stages of placental involvement the tissue viral loads likely decrease beyond the threshold of detection by in situ techniques, while morphologic features, including inflammation, necrosis, and fibrinoid deposition, still persist. Thus, intervillositis observed by us in the severely ill patient on ECMO (Figure 3) was highly suspicious for the late stage of the infection with features of “burned out” inflammation with marked necrosis of syncytiotrophoblast and encasement of chorionic villi by dense fibrinoid material causing ischemic changes in the villous cores. Interestingly, the immunohistochemical staining for C4d was similar to that described by Libbrecht et al. [9] in their case of SARS-CoV-2 intervillositis; however, as the authors noted, the C4d staining is not specific and had also been described in the so-called idiopathic intervillositis [20]. Thus, placental involvement could not be definitively confirmed in the case of the most severe COVID-19 in our cohort.

In sum, our study showed a very low level (about 1%) of definitive placental involvement in SARS-CoV-2 infected parturient women and this involvement did not correlate with the disease severity, as it was observed in one asymptomatic and one mildly symptomatic cases, while in the group of six patients with severe COVID-19 there was only one case of possible/early placental involvement and one case demonstrating an organizing stage of intervillositis, suspicious for a late stage in the evolution of the infectious lesion. These findings suggest that placental involvement is transient in the course of the infection and our ability to detect the virus in the placental tissue may be influenced by the time between the peak of tissue viral load and delivery.

Our data confirmed previous reports [14,15,21,22] on the rarity of perinatal involvement in SARS-CoV-2 as all tested (87.3%) neonates born to the SARS-CoV-2 positive women were negative for the virus and no perinatal complications or hospital stays have been attributed to the MTCT of the infection. Three identified cases of the fetal loss among 197 infected parturient women were likely unrelated to SARS-CoV-2 since available fetal tissues were negative for the virus and pathology other than SARS-CoV-2 infection was present to explain the demise in each of the three cases.

Our analysis of four perinatal autopsies in women negative by SARS-CoV-2 RT-PCR but positive for COVID-19 serology showed that in three cases the causes of fetal demise (cord accident, immune hydrops, and pulmonary hypoplasia) were unlikely related to the prior maternal infection during pregnancy. In one case, however, the direct cause of the near term IUFD was not apparent with only one known risk factor (maternal obesity). Positive COVID-19 serology, which was also detected in the fetal serum, raised a question of a fetal infection. Unfortunately, the Elecsys^®^ Roche assay which we used does not differentiate between IgG and IgM antibodies; thus, it is not possible to tell whether the test positivity was entirely due to the passage of IgG antibodies from the mother or whether a fraction of IgM antibodies was also present in the fetal serum. The latter would have indicated a fetal infection, since IgMs do not cross placenta. However, due the test design, the fetal infection remained indeterminate. In this case the endothelium of fetal lungs showed immune reaction with the antibody against SARS-CoV-2 nucleocapsid protein; however, an active SARS-CoV-2 infection due to a replicating virus was ruled out by the negative ISH and RT-PCR in pulmonary tissue homogenates. The findings were unusual prompting us to question the observed staining as an unspecific reaction or artefact; however, we have not observed an artificial staining with this antibody in numerous other cases of placentas and neonatal lungs (Figure 4C,D).

Interestingly, staining of the endothelium of villous capillaries was reported by Schwartz et al. [12] in two placentas, including one case of a COVID-19 infection remote from delivery when no syncytiotrophoblastic staining was observed. A positive staining of placental Hoffbauer cells was also seen in their cases and the authors hypothesized that the antibody may have reacted with persistent viral particles engulfed by macrophages and sequestered in the endothelium.

If this hypothesis is accepted and fetal endothelial cells can internalize the virus or its debris, the anti-nucleocapsid staining of the pulmonary endothelium in our case of the fetus positive for COVID-19 serology would be consistent with the history of MTCT of SARS-CoV-2 sometime during pregnancy. Thus, with all the limitations, this single case is the best candidate for the MTCT of SARS-CoV-2 in our one-year experience. Interestingly, the virus has been recently demonstrated in the fetal tissues by RT-PCR and immunofluorecence IHC in a case of IUFD at 34 weeks to a woman with a mild infection [23]. More data are needed to develop consensus criteria for the definitive, probable and possible diagnoses of SARS-CoV-2 infection and MTCT on autopsy material.

Recent study demonstrated the expression of the main SARS-CoV-2 cellular entry factors in villous trophoblast in term placentas, explaining why this cellular layer gets attacked by the virus [24]. The individual variability of these expression levels may be responsible for the different susceptibility to placental infection among women. Because the MTCT and fetal involvement are even rarer than the placental infection, we agree that the mechanisms underlying placental defense against SARS-CoV-2 likely involve post entry viral processing, potentially including phagocytosis by Hofbauer cells and engulfment by fetal endothelium. Further studies are necessary to understand the role of different components of the placenta in defending the offspring from SARS-CoV-2.

## Data Availability

Not applicable.
